# Management of Root Rot (*Rhizoctonia solani* Kühn) of Common Bean Using Host Resistance and Consortia of Chemicals and Biocontrol Agents

**DOI:** 10.3390/biology14030235

**Published:** 2025-02-25

**Authors:** Pratikshya Parajuli, Ritesh Kumar Yadav, Hira Kaji Manandhar, Megha N. Parajulee

**Affiliations:** 1 Department of Plant Pathology, Agriculture and Forestry University, Chitwan, Rampur, Nepal; 2 Department of Entomology, Texas A&M University, Texas A&M AgriLife Research and Extension Center, Lubbock, TX 79403, USA

**Keywords:** biocontrol agents, common bean (*Phaseolus vulgaris* L.), chemical control, landraces, root rot (*Rhizoctonia solani*)

## Abstract

Rhizoctonia root rot and web blight caused by *Rhizoctonia solani* Kühn is one of the destructive diseases of the common bean (*Phaseolus vulgaris* L.). A two-year study was conducted (2019–2021) to evaluate the common bean landraces and genotype, biocontrol agents, and chemical fungicides against the pathogen. Nine landraces from high-altitude agriculture in the Jumla district in western Nepal (29.27443900, 82.19332200; 2540 m msl) and one genotype of commercial common bean from the sub-tropical Chitwan district in central Nepal (27.65186564, 84.34869288; 256 m masl) were evaluated for resistance against the pathogen under screenhouse and field conditions. All were found highly susceptible to the pathogen and the highest lesion length (3.02 cm) was measured in the *Rato Male Simi* followed by *Rato Sano Chhirke Simi* (2.40 cm) in Chitwan conditions. Three chemical fungicides—SAAF™ (carbendazim 12% + mancozeb 63% WP), Vitavax^®^ (carboxin 37.5% + thiram 37.5% DS), and Nativo^®^ (tebuconazole 50% + trifloxystrobin 25% *w*/*w* (75 WG)—and three biocontrol agents—*Trichoderma viride* (10^9^ cfu/mL), *Pseudomonas fluorescens* (10^9^ cfu/mL), and *Bacillus subtilis* (10^8^ cfu/mL)—were evaluated as seed and soil treatments in fourteen different combinations against *R. solani*. SAAF™ and Vitavax^®^ were found highly effective as they completely suppressed the incidence of pre-emergence damping off. SAAF™ was equally effective followed by Vitavax^®^ and Nativo^®^ in suppressing post-emergence damping off. On the other hand, the highest disease incidence (26.63%) was recorded in treatment where seed treatment with *Pseudomonas fluorescens* formulation at the rate of 10 g per kg of FYM slurry along with soil application with *Pseudomonas fluorescens* formulation at the rate of 10 kg per ha with 50 kg FYM was applied, whereas the lowest disease incidence (4.96%) occurred in SAAF™ for both seed and soil applications. Highest grain yield (4125 kg/ha) was recorded on Nativo^®^ where seed treatment at the rate of 1.5 g per kg along with soil drenching at the rate of 1.5 g per liter was used. Thus, it can be concluded that the best control for pre-emergence damping off was achieved by SAAF™ where seed treatment at the rate of 2 g per kg along with soil drenching at the rate of 2 g per liter and Vitavax^®^ as seed treatment at the rate of 2 g per kg along with soil drenching at the rate of 2 g per liter was used, while the best control of post-emergence damping off was achieved with combined seed treatment and soil treatment with SAAF™. There was no incidence of web blight throughout the growing period. Our findings could be useful in devising strategies for the integrated management of *R. solani* causing root rot disease in common beans.

## Simple Summary

The common bean (*Phaseolus vulgaris* L.), *Simee* in vernacular Nepali, is grown during summer in the mid and high hills of Nepal. It is an important dietary source of carbohydrates, proteins, vitamins, fibers, and essential minerals. Despite their popularity, the productivity of beans is at suboptimum level due to various biotic stress factors, including diseases. Among them, damping off and root rot caused by *Rhizoctonia solani* is one of the major limiting factors in bean production. This research aimed to identify resistance sources in the common bean and to evaluate the efficacy of commonly used biocontrol agents and selected fungicides under screenhouse and field conditions to help identify better management options for *Rhizoctonia solani*. Our research indicated that among the tested ten bean genotypes/landraces, none was found resistant to the disease. Similarly, none of the three biocontrol agents evaluated (*Trichoderma viride*, *Bacillus subtilis*, and *Pseudomonas fluorescens*) were effective against *R. solani*. Chemical fungicide SAAF™ (carbendazim 12% + mancozeb 63% WP) (Mumbai, India) was the most effective in controlling damping off and root rot diseases followed by Vitavax^®^ (carboxin 37.5% + thiram 37.5% DS) (Haryana, India) and Nativo^®^ (tebuconazole 50% + trifloxystrobin 25% WG) (Karnataka, India).

## 1. Introduction

The common bean (*Phaseolus vulgaris* L.) is considered one of the most important leguminous crops worldwide including Nepal. It is one of the most important crops comprising 50% of the world legume consumption [1,2]. It is characterized as an important dietary source of carbohydrates, proteins, vitamins, fibers, and essential minerals [3]. In addition, *Phaseolus vulgaris* is considered as reliable source of income generation at local level among the small-scale subsistence farmers of high mountainous regions in Nepal. The common bean is a tender, warm-season vegetable crop which requires a mean air temperature of 20 °C to 25 °C for its optimum growth and development [4]. There are two types of beans according to growth habit: pole type and bush type. Pole type varieties are tall, indeterminate in growth, and require support, whereas bush type varieties are dwarf and are more popular because of their compactness, ease of harvest, and short duration (60–70 days). In addition to numerous valuable agronomic characteristics of the common bean, it holds a prominent place as a unique commodity. Nepal, being the 10th richest country in agro-biodiversity in Asia and 31st in the world, provides a significant space for geographical indications (GIs) of major crops like the common bean [5].

Common beans are highly susceptible to several diseases attacking the aerial and underground parts [6,7,8]. Among them, damping off and root rot caused by *Rhizoctonia solani* is considered one of the major limiting factors in bean production. Economic losses due to the disease have often been estimated to be 100% among the bean growers in the context of Nepal [9], although losses can vary greatly from one field to another depending on cropping history and environment. In addition to root and stem rot of the bean, the fungus also can cause pre-emergence and post-emergence damping-off where *R. solani* anastomosis group 4 (Ag-4) is more commonly involved. The pathogen is more prevalent in cool and humid environments [10] and can cause severe disease in various crops including vegetables (bean, lettuce), cereals, cotton, ornamental plants, and forest trees [11,12,13,14].

The production and productivity of common beans are mainly constrained by low yield and susceptibility to diseases. Despite the fourth rank of grain legumes in terms of area and production in Nepal, a notable production decline in production and productivity is reported in recent years [15] due to disease epidemics. Research findings revealed that one of the potential factors that contribute to the lower yield of legume crops including beans is their increasing vulnerability to a range of diseases mostly caused by fungi [9] and among the major fungal diseases, *Rhizoctonia solani* is more destructive due to its persistence in soil.

In view of the economic importance of the bean crop and effect of the disease on yield and quality, there is a critical need for detailed research on effective management options. The disease has become a significant production risk due to several reasons including epidemiology, its persistent nature, and evolution of new strains against available fungicides. So, proficient and effective use of chemicals, bio-control agents, and resistant varieties and landraces may have potential to control the rhizoctonia root rot of bean. Integrated disease management combines multiple control measures such as chemical treatments, biological control agents, and resistant varieties to provide a comprehensive approach to disease management, increasing the effectiveness of disease control. Several researchers have found that the combined application of biocontrol agents along with organic amendments and fungicides is highly effective in controlling soil-borne as well as seed-borne plant pathogens during crop cultivation [16,17,18,19]. Moreover, use of resistant varieties is the most practical, economical, and eco-friendly approach for management of soil borne diseases [20]. To initiate breeding for disease resistance, identification of sources of resistance is imperative. However, at present, information on bean crop disease bionomics and the assessment of appropriate integrated disease management for the Nepal region is lacking. Therefore, this research aimed to identify resistance sources in the common bean through screening and to evaluate the efficiency of commonly used biocontrol agents and chemical fungicides under screenhouse and field conditions to help identify better management options for *Rhizoctonia solani*. Specifically, the study (i) examined elite bean genotypes and landraces against rhizoctonia root rot under field and screenhouse conditions, and (ii) evaluated various management options for the disease using host resistance, chemicals, and biocontrol agents under field conditions.

## 2. Material and Methods

Experiments were conducted at the screenhouse and research field of the Agriculture and Forestry University (AFU) campus in Rampur, Chitwan, Nepal from November 2019 to April 2021.

### 2.1. Plant Material, Biocontrol Agents, and Fungicides

#### 2.1.1. Source of Common Bean Seeds

Nine bean landraces including *Fusro Chhirke Simi*, *Kalo Lamo Simi*, *Rato Male Simi*, *Kalo Sano Simi*, *Khairo Sada Simi*, *Rato Sano Chhirke Simi*, *Rato Lamo Simi*, *Pahelo Besare Simi*, and *Rato Sano Simi* were obtained from Agriculture Research Station, Bijaynagar, Jumla of the Nepal Agricultural Research Council (NARC), and one genotype Rato Chhirke Simi collected locally from Chitwan was included as a susceptible check. The seeds of these landraces and genotypes varied in seed size and color for easy discrimination and were the most elite lines developed by NARC in terms of their production and productivity. The names of these landraces/genotypes were derived from their phenotypic characteristics and local dialects.

#### 2.1.2. Source of the Pathogen Isolate and Inoculum Preparation

The fungus *Rhizoctonia solani*, isolated from a root rot sample of bean showing damping off symptoms grown in a nearby vegetable farm in Sharadanagar, Chitwan, was used. The isolate was confirmed for its pathogenicity and maintained in PDA slants and stored at 5 °C. For inoculation, the pathogen was multiplied in wheat grains.

#### 2.1.3. Isolation Technique for Rhizoctonia Root Rot and Identification of *R. solani*

The conventional technique was used for the isolation of the pathogen. Tissue samples from common bean plants exhibiting root rot symptoms were evaluated and processed for isolation. The roots of infected plants were first washed with tap water to remove soil. Small portions of root and stem sections near visible necrotic areas or lesions were then excised. The plant tissue then underwent surface sterilization through two consecutive treatments, each lasting one minute: first with 1% sodium hypochloride (NaOCl) solution followed by sterile distilled water. Then, the sterilized root and stem segments were placed in petri dishes containing PDA. The plates were then incubated at 25 ± 1 °C for 2 days. The growing mycelia were picked with an inoculation needle, transferred on PDA slants, and incubated at 25 ± 1 °C for seven days. This similar procedure was also followed by [21]. For the purpose of identification, the fungal isolates were identified based on species-specific salient characteristics using morphological keys [22,23]. Also, the examination of cultural characteristics revealed that the colony color of the isolate varied from light to dark-brown in PDA culture plates. The isolate used in our experiment showed close agreement with [24] where the key to differentiate mycelium of multinucleate rhizoctonia species has been described. Identification of *R. solani* was further verified by the standard conventional plant pathological technique.

#### 2.1.4. Source of Biological Agents and Chemical Fungicides

One fungal biocontrol agent, *Trichoderma viride* (10^9^ cfu/mL), two bacterial agents, *Pseudomonas fluorescens* (10^9^ cfu/mL) and *Bacillus subtilis* (10^8^ cfu/mL), and three chemical fungicides, SAAF™ (carbendazim 12% + mancozeb 63% WP) (Mumbai, India), Vitavax^®^ (carboxin 37.5% + thiram 37.5% DS) (Haryana, India), and Nativo^®^ (tebuconazole 50% + trifloxystrobin 25% *w*/*w* (75 WG) (Karnataka, India), were evaluated as seed and soil treatments. Descriptions of these biocontrol agents and chemical fungicides and their sources are provided in Table 1.

#### 2.1.5. Isolation and Identification of the Pathogen

**Figure 1:**
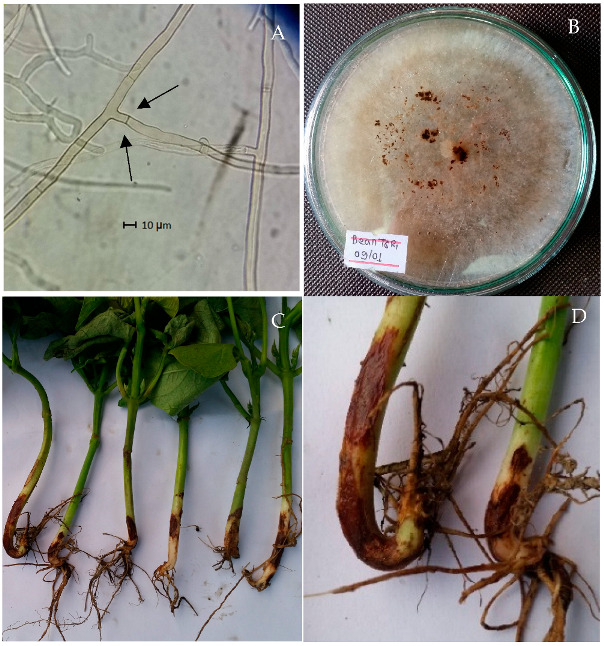
Typical features of *R. solani* under compound microscope (100×), septate hypha with right-angle branching and constriction at the point of branching, septum close to the origin indicated by arrows (**A**), mycelial growth of *R. solani* in PDA plate (**B**), and common bean root rot symptoms (**C**,**D**).

*Rhizoctonia solani* isolated and used in the experiments had multinucleated cells and produced white to deep brown mycelium on PDA medium (Figure 1). Branching near the distal septum of young hyphae with slight constriction and formation of septa near to the point of origin of branches (Figure 1A) was the diagnostic characteristics for *R. solani* [25].

### 2.2. Screenhouse and Field Experiments to Identify the Resistant Landraces and Genotype

The trials were carried out in the screenhouse of Agriculture and Forestry University, Rampur, Chitwan. For the experiment, the same set of nine bean landraces and one genotype was used in screenhouse and field experiments under artificially inoculated conditions. The experiment was laid out in a completely randomized design (CRD) with four replications. For screenhouse experiments, plastic pots (15 cm diameter) with 1 kg capacity were filled with sterilized field soil and bean seeds were sown (2 seeds per pot). Fifteen-day-old seedlings of test beans were inoculated by applying 2 g culture of *R. solani* around the collar region after making a small injury using a sterilized pin. Moist cotton was placed over the inoculated area to maintain moisture for facilitating infection by the pathogen. The inoculated plants were covered with a polypropylene sheet sprinkled with water to create congenial conditions for the pathogen. Holes were made in the sheet for aeration.

For field experiments, the size of individual plot was 3 m^2^. Field plot was prepared by ploughing, harrowing, and leveling. Farmyard manure (FYM) 4 kg per plot plus N-P_2_O_5_-K_2_O 80-60-40 kg per hectare was applied. Half of the nitrogen was applied as basal dose, and the remaining half was applied two months after seeding. Intercultural operations (hoeing, weeding, and earthing up) were carried out as a standard practice [15]. The experiment was conducted under natural epiphytotic conditions in a field plot where the disease had occurred in the previous season.

Root rot was recorded and disease incidence (number of diseased plants out of number of plants observed × 100) was calculated. The bean landraces and genotype were categorized following the disease incidence (Figure 1) scale [26]: 0–1% *Resistant*, 1.1–10 *Moderately Resistant*, 10–25% *Moderately Susceptible*, and 25.1–50% *Susceptible*.

### 2.3. Field Evaluation of Fungicides and Biocontrol Agents Against Rhizoctonia Root Rot

The field experiment was carried out at AFU horticulture farm to investigate the effect of biological and chemical treatments on controlling root rot disease. The experiment was deployed in a randomized complete block design with three replications. The size of each individual plot was 3 m^2^. The field plot was prepared by ploughing, harrowing, and leveling as described previously. FYM 4 kg per plot plus N-P_2_O_5_-K_2_O: 80-60-40 kg per hectare was applied. Half of the nitrogen was applied as basal dose, and the remaining half was applied two months after seeding. Intercultural operations (hoeing, weeding, and earthing up) were carried out as a standard practice [15]. Fourteen different treatment combinations were evaluated in a field using *Rato Chhirke Simi* as a host under artificially inoculated conditions (Table 2).

#### 2.3.1. Preparation of Seed for Experiment

Seeds used in the experiment were treated with chemical fungicides and biocontrol agents as assigned to their respective treatments (Table 1 and Table 2). *Rato Chhirke Simi*, a commercial genotype of Chitwan, was collected from the farmer’s field. The identification of the genotype was established based on the local name given by farmers. Substrates used for the growth and multiplication of the biocontrol agents were farmyard manure, spent mushroom substrate, and vermicompost.

#### 2.3.2. Artificial Inoculation Under Field Conditions

Plants were artificially inoculated uniformly and consistently across all treatment plots with mass culture suspension. Suspension was prepared by mixing 4 g of mass culture in 1 L of water and sprayed over the plant’s surface with the help of a sprayer. Foliar application of inoculation was carried out on 1-month-old plants.

### 2.4. Data Recording and Disease Assessment

Field observation and recording on disease symptoms was conducted after the initiation of visible symptoms of reddish-brown canker on the root region on any of the treatment plots. Plants showing typical symptoms were taken into consideration and it was recorded. The percentage of root rot incidence at the pre-emergence and post-emergence of growth stages was investigated and calculated 15 and 22 days after sowing, respectively. Plants showing symptoms were observed and counted considering the whole plot. Incidence of collar rot was assessed and calculated under screenhouse and field conditions by observing the root rot symptoms at the collar region. The number of diseased plants was counted considering the single plot and disease incidence was calculated by using the following formula,

$$\mathrm{Disease}\;\mathrm{incidence}\;\%\;=\;\frac{\mathrm{Number}\;\mathrm{of}\;\mathrm{diseased}\;\mathrm{plants}}{\mathrm{Total}\;\mathrm{number}\;\mathrm{of}\;\mathrm{plants}\;\mathrm{observed}}\;\times \;100$$

There was no occurrence of web blight during the research period. The obtained yield was estimated for each treatment as kg/plot at the end of the experiment.

### 2.5. Statistical Analysis

The recorded data were tabulated in an Excel data sheet and were subjected to various statistical analyses using the reference of Gomez and Gomez (1984) [27]. The data were processed, and final analyses were carried out using R-stat Version 4.0.5 (31 March 2021), “Shake and Throw”. Based on ANOVA results, Duncan’s multiple range test was performed for mean comparison at the *p* = 0.05 level of significance.

## 3. Results

### 3.1. Response of Landraces and Genotype Against Root Rot

All nine landraces and one genotype of common bean evaluated were found highly susceptible to the disease with 96 to 100 percent incidence of root rot under natural epiphytotic conditions in the field and with 100% incidence under inoculated conditions in the screenhouse (Table 3). Also, the lesion length size varied significantly under screenhouse conditions. Under screenhouse conditions, the lesion length ranged from 1.35 cm (*Kalo Lamo Simi*) to 3.02 cm (*Rato Male Simi*) evaluated against the root rot. All the tested landraces and genotypes were found highly susceptible to the disease with 100 percent incidence of root rot.

### 3.2. Evaluation of Different Chemical Fungicides and Biocontrol Agents Under Field Conditions

All the chemical fungicides were found effective in reducing the incidence of pre-emergence and post-emergence damping off as compared to untreated control. However, the results revealed that treatments varied in their performance in reducing the disease incidence (Table 4). SAAF™ (seed treatment at the rate of 2 g per kg + soil drenching at the rate of 2 g per liter) and Vitavax^®^ (seed treatment at the rate of 2 g per kg + soil drenching at the rate of 2 g per liter) were found highly effective in reducing the incidence of pre-emergence damping off to zero percent as compared to control and were statistically superior to other treatments followed by Nativo^®^ (tebuconazole 50% + trifloxystrobin 25% WG). All three biocontrol agents, *Pseudomonas fluorescens* (10^9^ cfu/mL), *Bacillus subtilis* (10^8^ cfu/mL), and *Trichoderma viride* (10^9^ cfu/mL), were less effective than chemical fungicide options. In the case of post-emergence damping off, SAAF™ was found highly effective and was significantly superior to other treatments followed by Vitavax^®^ and Nativo^®^. Data for pre-emergence and post-emergence damping off were taken 15 and 22 days after sowing, respectively.

The data for root rot infected plants showed that the incidence percentage for biocontrol agents ranged from 4.96 to 26.63 (Table 4). Status of incidence of pre-emergence damping-off was such that treatments were significantly different (*p* < 0.05) in terms of their prevalence (Table 4). Accordingly, the highest prevalence was with T14 (untreated control). Similarly, treatments, T2 (*Pseudomonas fluorescens*: seed treatment using 10 g per kg of farm yard manure slurry + soil treatment using 10 kg per ha with 50 kg FYM), and T4 (*Bacillus subtilis*: seed treatment using 10 g per kg vermicompost slurry + soil treatment using 10 kg per ha with 50 kg vermicompost), and T7 (*Trichoderma viride*: seed treatment at the rate of 10 g/kg vermicompost slurry + soil treatment at the rate of 10 kg/ha with 50 kg vermicompost) were statistically similar but numerically highest among treatments. Although non-significant, T10 (Nativo^®^: seed treatment at the rate of 1.5 g per kg + soil drenching at the rate of 1.5 g per liter) suppressed disease incidence to zero while T11 (Nativo^®^: seed treatment at the rate of 1.5 g per kg + foliar spray at the rate of 1.5 g per liter) resulted in some low disease incidence (Table 4). Overall, seed treatment and soil drenching in combination with Nativo^®^, SAAF™, or Vitavax^®^ were equally effective in reducing pre-emergence damping off (Table 4).

For post-emergence damping off, there was an inconsistent treatment effect in reducing disease incidence. Accordingly, the highest incidence was for treatment T4 (*Bacillus subtilis*: seed treatment using 10 g per kg of vermicompost slurry + soil treatment using 10 kg per ha with 50 kg vermicompost) and control whereas they were statistically similar (*p* > 0.05) to most of the other treatments, except T1 (*Pseudomonas fluorescens*: seed treatment using 10 g per kg of vermicompost slurry + soil treatment using 10 kg per ha with 50 kg vermicompost) and T10 to T13 in terms of rendering a similar effect (Table 4).

Effects of treatment on incidence of root rot also revealed that treatment responses varied across the fourteen treatment combinations (*p* < 0.05), but the variation in data revealed less statistical discrimination across treatments (Table 4).

### 3.3. Grain Yield

Effect of biocontrol agents and chemical fungicides on grain yield (kg/ha) of the common bean under field conditions at Rampur, Chitwan, revealed that the highest grain yield of bean was obtained from treatment 11 (Nativo^®^: seed treatment at the rate of 1.5 g per kg + foliar spray at the rate of 1.5 g per liter), (Table 4). This was, however, statistically similar (*p* > 0.05) to treatment T12 (SAAF™: seed treatment at the rate of 2 g per kg + soil drenching at the rate of 2 g per liter) and treatment T13 (Vitavax^®^: seed treatment at the rate of 2 g per kg + soil drenching at the rate of 2 g per liter) (Table 4). The control treatment produced the least grain yield which was about 30% lower than the highest grain yield (Table 4). Similarly, treatments with seed treatments along with soil treatment in combination with FYM and SMS (T2, T3, T4, T6 to T8) also produced lower yields compared to T11 and T12. Treatments *Trichoderma viride* (seed treatment using 10 g per kg of vermicompost + soil treatment using 10 kg per ha with 50 kg SMS) and *Pseudomonas fluorescens* (seed treatment using 10 g per kg of vermicompost slurry + soil treatment using 10 kg per ha with 50 kg vermicompost) produced the lowest amount of grain yield which was numerically lower than for control (Table 4).

## 4. Discussion

Root rot and web blight of the common bean caused by *R. solani* is the most destructive among the fungal diseases. It is challenging to manage the disease because the pathogen lives in the soil and combines high saprophytic competitiveness with a wide host range [28,29]. While biocontrol agents have been explored as a potential solution, their effectiveness in the field has been limited, and this can be attributed to several factors including strain suitability, environmental factors, chemical incompatibility, and others. It should be noted that the biocontrol agents such as *Trichoderma*, *Pseudomonas*, and *Bacillus* used in our study have limitations when applied in the field on a large scale [30]. Some of the reasons for this limitation include temperature restrictions [30] and incompatibility with chemicals applied in integrated controls [31].

In our study, biocontrol agents were not found effective. As reported in the literature, successful control of the disease depends on interference with the infection process such as initiation of the primary infection by soilborne inocula or possible further development of secondary infection [32]. Therefore, ineffectiveness of the tested biocontrol agents in our study is likely due to the hindrance of the infection process manifested by temperature restrictions [31] or may be the result of an ineffective strain.

While biocontrol agents may have limitations, it is crucial to explore alternative strategies for the management of these diseases. Using resistant genotypes is proven to be an important measure for integrated control of this disease in several crops [33]. The use of resistant cultivars would result in improved stands and higher yields in crops. Furthermore, resistance to disease or any disease complex would be helpful in enhancing the effectiveness of seed and soil treatments thereby improving the development of more effective methods of integrated disease control [34]. Also, landraces are of great importance as there could be an opportunity to select a high-yielding, disease-resistant, and locally accepted variety [35]. Experimenting with landraces also provides the possibility of producing new varieties with combined traits transferred from different varieties through breeding methods as reported [35].

In our experiment under screenhouse conditions, 100 percent disease incidence occurred, but there was variation in lesion length. The variation in lesion length, ranging from 1.35 cm for *Kalo Lamo Simi* to 3.02 cm for *Rato Male Simi*, suggests that there are inherent differences in the response of these landraces to *R. solani* infection. However, the variation in lesion length indicates that while all the landraces were infected, some might have possessed a degree of tolerance or partial resistance that limited the extent of damage caused by the pathogen. The landraces with shorter lesion length [*Kalo Lamo Simi* (1.35 cm), *Kalo Sano Simi* (1.40 cm), *Rato Chhirke Simi* (1.80 cm)] can be valuable for breeding programs aiming to develop bean varieties with improved resistance to root rot. Similarly, during the screening experiment conducted under field conditions, a 100 percent disease incidence was reported. No single landrace or genotype was found resistant. Several researchers have reported the existence of resistance in certain genotypes. The authors of [36] reported six lines showing resistant reaction. Among the lines used in their experiment, IC-272638, IC-258275, IIHR-909, VRF-3-2, and Arka Komal were found resistant to the disease in both natural and artificially inoculated conditions. Likewise, Refs. [37,38,39] screened several accessions and reported some resistant lines.

Refs. [40,41,42] reported that lesion length on the collar region, plant height, plant weight, and the percent of disease incidence are the most important parameters for determination of disease severity in most studies. It was also shown that with the development of disease in susceptible plants, the length of the lesions increased, and the weight of the plant decreased gradually. We have also followed a similar approach of starting with a certain number of strains/accessions to screen the disease. Accordingly, a total of nine landraces and one genotype were identified as susceptible and none of them were found resistant against the disease.

Our research also evaluated the consortium of safer chemical fungicides and biocontrol agents in managing the root rot disease under field conditions. It is important because besides eradicative action and other means of disease management, chemical toxic barriers against pathogens are the unavoidable means of controlling many plant diseases. Also, fungicides dominate as the most common method for controlling *R. solani* [43,44,45]. In our study, we used SAAF™, Nativo^®^, and Vitavax^®^ as seed treatment and soil drenching. In the present investigation, plots treated with the fungicides SAAF™, Nativo, and Vitavax^®^ as seed treatment and soil drenching were all effective in controlling disease in comparison to the biocontrol agents used. The present findings are in close agreement with those earlier reported by [46] who also reported the effectiveness of seed treatment with Vitavax^®^ imparting the lowest percent disease index and higher yield of lentil against wilt/root rot complex. Our findings match well to the report of other researchers managing the root rot of large cardamom [47]. Equally, the results obtained with SAAF™ and Vitavax^®^ are in conformity with the findings of [48] as the authors reported SAAF™ as the most effective fungicide followed by carbendazim (98.9%), Vitavax^®^ (98.2%), propiconazole (74.8%), and hexaconazole (72.9%) against *R. solani* causing sheath blight of rice. Similarly, Ref. [49] reported seed treatment with *T. viride* + Vitavax^®^ + *Rhizobium* to show superiority over other treatments with respect to increasing germination, plant vigor, root nodules, and yield and decreasing plant mortality and disease intensity in urd and mung bean against web blight disease.

Nativo^®^ was found to record numerically higher grain yield as compared to other fungicides. This may be due to the beneficial role of trifloxystrobin in combination with tebuconazole for yield improvement. A similar observation was reported in rice where improved root length and total dry matter along with increased panicle length, 1000-grain weight, and grain yield were found [50]. On the other hand, biological control is mainly concentrated on eradication and or/management of pathogens through the activity of other microorganisms. In this line, several research findings are reported considering different strains and species such as those related to strains of the *Trichoderma* spp. [51,52,53], *Bacillus* spp. [54,55,56,57], and *Pseudomonas* spp. In the present study of management of root rot under field conditions, the biocontrol agents, *Pseudomonas fluorescens* from Agricare Nepal, Chitwan, *Bacillus subtilis* from NPDA (Nepal Plant Disease and Agro Associates), Kathmandu, and *Trichoderma viride* from Agricare, Chitwan, did not show significant effect against the pathogen. The reason for the lack of effectiveness of these biocontrol agents against *R. solani* in our study is not apparent, but we speculate that factors including the mode of action, variation in strains of pathogens, environmental conditions such as soil temperature, soil moisture status, pH value, nutrient availability, and interactions with indigenous soil microbes rendered them ineffective [58].

## 5. Conclusions

The present study found higher efficacy of chemical fungicides such as SAAF™ (carbendazim 12% + mancozeb 63% WP) for controlling the root rot disease followed by Vitavax^®^ (carboxin 37.5% + thiram 37.5% DS) and Nativo^®^ (tebuconazole 50% + trifloxystrobin 25% WG) in comparison to biocontrol agents (*Trichoderma viride*, *Bacillus subtilis*, and *Pseudomonas fluorescens*), whereas Nativo^®^ was effective in increasing the grain yield in comparison to other fungicides and biocontrol agents. Thus, it can be inferred that these fungicides can be included in controlling rhizoctonia root rot of common beans along with enhancing the grain yield of beans. Similarly, evaluation of 10 selected landraces and genotypes of common beans in the screenhouse as well as in the field led us to identify all of them as highly susceptible to the disease.

## Figures and Tables

**Table 1:** Trade name, chemical name, and manufacturer/source of biocontrol agents and chemical fungicides evaluated.

Trade Name	Chemical Name	Manfacturer/Source
SAAF^TM^	carbendazim 12% + mancozeb 63%	United Phosphorous Limited Khar, Mumbai, India E-mail: customer.care@upl-ltd.com
Vitavax^®^	carboxin 37.5% + thiram 37.5%	Dhanuka Agritech Limited Gurugram, Haryana, India E-mail: headoffice@dhanuka.com
Nativo^®^	tebuconazole 50% + trifloxystrobin 25% *w*/*w* WG (75 WG)	Agriplex Bengaluru, Karnataka, India E-mail: support@agriplexindia.com
*Bacillus subtilis*	*Bacillus subtilis* spores content: 1 × 10^8^ cfu/mL	Nepal Plant Disease and Agro Associates (NPDA), Nepal Balaju, Kathmandu, Nepal E-mail: nepalpda@gmail.com
*Trichoderma viride*	*Trichoderma viride* spores content: 1 × 10^9^ cfu/mL	Agricare Nepal Bharatpur, Chitwan, Nepal E-mail: hello@agricare.com.np
*Pseudomonas fluorescens*	*Pseudomonas fluorescens* spores content: 10^9^ cfu/mL	Agricare Nepal Bharatpur, Chitwan, Nepal E-mail: hello@agricare.com.np

**Table 2:** Fourteen treatment combinations of chemical insecticides and biocontrol agents evaluated under field conditions against *R. solani.*

Treatment ID	Treatment Details
T1	Seed treatment (ST) with *Pseudomonas fluorescens* formulation (10^9^ cfu/mL) at the rate of 10 g per kg of vermicompost slurry + soil treatment (SoT) with *Pseudomonas fluorescens* formulation (10^9^ cfu/mL) at the rate of 10 kg per ha with 50 kg vermicompost
T2	Seed treatment (ST) with *Pseudomonas fluorescens* formulation (10^9^ cfu/mL) at the rate of 10 g per kg of farm yard manure (FYM) slurry + soil treatment (SoT) with *Pseudomonas fluorescens* formulation (10^9^ cfu/mL) at the rate of 10 kg per ha with 50 kg of farmyard manure (FYM)
T3	Seed treatment (ST) with *Pseudomonas fluorescens* formulation (10^9^ cfu/mL) at the rate of 10 g per kg of vermicompost slurry + soil treatment (SoT) with *Pseudomonas fluorescens* formulation (10^9^ cfu/mL) at the rate of 10 kg per ha with 50 kg of spent mushroom substrate (SMS)
T4	Seed treatment (ST) with *Bacillus subtilis* (10^8^ cfu/mL) at the rate of 10 g per kg of vermicompost slurry + soil treatment (SoT) with *Bacillus subtilis* (10^8^ cfu/mL) at the rate of 10 kg per ha with 50 kg vermicompost
T5	Seed treatment (ST) with *Bacillus subtilis* (10^8^ cfu/mL) at the rate of 10 g per kg of farm yard manure (FYM) slurry + soil treatment (SoT) with *Bacillus subtilis* (10^8^ cfu/mL) at the rate of 10 kg per ha with 50 kg of farm yard manure (FYM)
T6	Seed treatment (ST) with *Bacillus subtilis* (10^8^ cfu/mL) at the rate of 10 g per kg of farm yard manure (FYM) slurry + soil treatment (SoT) with *Bacillus subtilis* (10^8^ cfu/mL) at the rate of 10 kg per ha with 50 kg spent mushroom substrate (SMS)
T7	Seed treatment (ST) with *Trichoderma viride* formulation (10^9^ cfu/mL) at the rate of 10 g per kg of vermicompost slurry + soil treatment (SoT) with *Trichoderma viride* formulation (10^9^ cfu/mL) at the rate of 10 kg per ha with 50 kg vermicompost
T8	Seed treatment (ST) with *Trichoderma viride* formulation (10^9^ cfu/mL) at the rate of 10 g per kg of vermicompost slurry + soil treatment (SoT) with *Trichoderma viride* formulation (10^9^ cfu/mL) at the rate of 10 kg per ha with 50 kg of farm yard manure (FYM)
T9	Seed treatment (ST) with *Trichoderma viride* formulation at the rate of 10 g per kg of vermicompost slurry + soil treatment (SoT) with *Trichoderma viride* formulation at the rate of 10 kg per ha with 50 kg spent mushroom substrate (SMS)
T10	Seed treatment (ST) at the rate of 1.5 g per kg with Nativo^®^ (tebuconazole 50% + trifloxystrobin 25% WG) + soil drenching (SD) with Nativo^®^ (tebuconazole 50% + trifloxystrobin 25% WG) at the rate of 1.5 g per liter
T11	Seed treatment (ST) at ther rate of 1.5 g per kg with Nativo^®^ (tebuconazole 50% + trifloxystrobin 25% WG) + foliar spray (FS) with Nativo^®^ (tebuconazole 50% + trifloxystrobin 25% WG) at the rate of 1.5 g per liter
T12	Seed treatment (ST) with SAAF™ (carbendazim 12% + mancozeb 63% WP) at the rate of 2 g per kg + soil drenching (SD) with SAAF™ (carbendazim 12% + mancozeb 63% WP) at the rate of 2 g per liter)
T13	Seed treatment (ST) with Vitavax^®^ (carboxin 37.5% + thiram 37.5% DS) at the rate of 2 g per kg + soil drenching (SD) with Vitavax^®^ (carboxin 37.5% + thiram 37.5% DS) at the rate of 2 g per liter
T14	Control—untreated check

**Table 3:** Incidence of root rot (*Rhizoctonia solani*) in common bean landraces and genotypes under natural epiphytotic (field conditions) and artificially inoculated conditions in the screenhouse at Rampur, Chitwan, 2020–2021.

Landraces	In the Field	In the Screenhouse		Disease Reaction
	Incidence of Collar Rot (%) ± SEM	Mean Lesion Length (cm) ± SEM	Incidence of Root Rot (%)	
*Fusro Chhirke Simi*	100 ^a^ ± 0	1.77 ^bc^ ± 0.17	100	HS
*Kalo Lamo Simi*	100 ^a^ ± 0	1.35 ^c^ ± 0.63	100	HS
*Rato Male Simi*	100 ^a^ ± 0	3.02 ^a^ ± 0.58	100	HS
*Kalo Sano Simi*	100 ^a^ ± 0	1.40 ^bc^ ± 0.42	100	HS
*Khairo Sada Simi*	100 ^a^ ± 0	1.92 ^bc^ ± 0.26	100	HS
*Rato Sano Chhirke Simi*	100 ^a^ ± 0	2.40 ^ab^ ± 0.32	100	HS
*Rato Lamo Simi*	100 ^a^ ± 0	2.07 ^abc^ ± 0.35	100	HS
*Pahelo Besare Simi*	100 ^a^ ± 0	1.90 ^bc^ ± 0.43	100	HS
*Rato Sano Simi*	100 ^a^ ± 0	2.07 ^abc^ ± 0.35	100	HS
*Rato Chhirke Simi*	96.10 ^b^ ± 2.42	1.80 ^bc^ ± 0.25	100	HS
LSD	2.27	1.032		
SEM (±)	0.24	0.11		
F-probability	*	*		
CV (%)	1.33	36.09		

* Indicates significance at 5% level, SEM (±) = Standard error of mean, LSD = Least significant difference, Values with same lowercase letter within column are not significantly different, CV% = Coefficient of variation, HS = Highly susceptible.

**Table 4:** Effect of biocontrol agents and chemical fungicides on the incidence of root rot and pre-emergence and post-emergence damping off due to *Rhizoctonia solani* along with the grain yield, Rampur, Chitwan, 2019–2021.

Treatments	Pre-Emergence Damping off (%) ± SEM	Post-Emergence Damping off (%) ± SEM	Root Rot (%) ± SEM	Grain Yield (kg/ha) ± SEM
T1: *Pseudomonas fluorescens:* seed treatment at the rate of 10 g/kg of vermicompost slurry + soil treatment at the rate of 10 kg/ha with 50 kg vermicompost	3.86 ^abc^ ± 0.57	3.86 ^bcdef^ ± 0.57	14.43 ^bc^ ± 2.94	2680.99 ^f^ ± 75.32
T2: *Pseudomonas fluorescens:* seed treatment at the rate of 10 g/kg FYM slurry + soil treatment at the rate of 10 kg/ha with 50 kg of FYM	7.20 ^a^ ± 1.47	7.2 ^abcde^ ± 1.48	26.63 ^a^ ± 3.46	3364.07 ^cde^ ± 251.29
T3: *Pseudomonas fluorescens:* seed treatment at the rate of 10 g/kg vermicompost slurry + soil treatment at the rate of 10 kg/ha with 50 kg SMS	4.96 ^abc^ ± 1.67	8.3 ^abc^ ± 0.99	15.86 ^bc^ ± 2.43	3080.99 ^cdef^ ± 399.52
T4: *Bacillus subtilis:* seed treatment at the rate of 10 g/kg vermicompost slurry + soil treatment at the rate of 10 kg/ha with 50 kg vermicompost	7.73 ^a^ ± 0.57	11.06 ^a^ ± 5.32	15.53 ^bc^ ± 3.36	3169.20 ^cdef^ ± 393.42
T5: *Bacillus subtilis:* seed treatment at the rate of 10 g/kg FYM + soil treatment at the rate of 10 kg/ha with 50 kg FYM	3.86 ^abc^ ± 2.41	4.96 ^bcdef^ ± 0.96	22.16 ^ab^ ± 5.81	2740.49 ^ef^ ± 260.85
T6: *Bacillus subtilis:* seed treatment at the rate of 10 g/kg FYM) slurry + soil treatment at the rate of 10 kg/ha with 50 kg SMS	4.93 ^abc^ ± 3.33	6.06 ^abcdef^ ± 0.54	13.30 ^bcd^ ± 1.91	3485.10 ^bcd^ ± 194.00
T7: *Trichoderma viride:* seed treatment at the rate of 10 g/kg vermicompost slurry + soil treatment at the rate of 10 kg/ha with 50 kg vermicompost	7.76 ^a^ ± 2.23	9.4 ^ab^ ± 1.10	14.43 ^bc^ ± 3.39	2992.79 ^def^ ± 273.38
T8: *Trichoderma viride:* seed treatment at the rate of 10 g/kg of vermicompost slurry + soil treatment at the rate of 10 kg/ha with 50 kg FYM	6.66 ^ab^ ± 1.67	7.76 ^abcd^ ± 1.47	7.2 ^cd^ ± 1.47	3362.20 ^cde^ ± 101.52
T9: *Trichoderma viride:* seed treatment at the rate of 10 g/kg of vermicompost + soil treatment at the rate of 10 kg per ha with 50 kg SMS	4.96 ^abc^ ± 1.67	7.16 ^abcde^ ± 0.57	7.16 ^cd^ ± 2.41	2631.77 ^f^ ± 447.08
T10: Nativo^®^: seed treatment at the rate of 1.5 g/kg + soil drenching at the rate of 1.5 g per liter	0 ^c^ ± 0	2.76 ^cdef^ ± 1.47	4.96 ^d^ ± 0.95	3189.71 ^cdef^ ± 211.41
T11: Nativo^®^: seed treatment at the rate of 1.5 g/kg + foliar spray at the rate of 1.5 g per liter	1.63 ^bc^ ± 0.95	2.2 ^def^ ± 1.48	7.2 ^cd^ ± 1.1	4125.09 ^a^ ± 238.61
T12: SAAF™: seed treatment at the rate of 2 g per kg + soil drenching at the rate of 2 g per liter	0 ^c^ ± 0	0.53 ^f^ ± 0.54	4.96 ^d^ ± 1.67	4016.37 ^ab^ ± 129.78
T13: Vitavax^®^: seed treatment at the rate of 2 g per kg + soil drenching at the rate of 2 g per liter	0 ^c^ ± 0	1.63 ^ef^ ± 0.96	7.73 ^cd^ ± 0.57	3694.32 ^abc^ ± 293.03
T14: Control	8.3 ^a^ ± 2.54	11.06 ^a^ ± 0.54	15.53 ^bc^ ± 1.47	2949.72 ^def^ ± 98.55
LSD	4.84	5.16	7.96	583.21
SEM (±)	0.44	0.47	0.73	53.61
F-probability	**	**	***	***
CV%	65.29	51.31	37.49	10.69

Means denoted by same lowercase letters within each column are not significantly different (** and *** indicate the probability at 1 and 0.1%, respectively); SEM = Standard error of mean, LSD = Least significant difference, CV% = Coefficient of variation, FYM = Farmyard manure, SMS = Spent mushroom substrate.

## Data Availability

Data are contained within the article in the form of tables and figures.
